# New tools for learning airway management: A report on the creation of an escape room and its development during an international airway course

**DOI:** 10.1097/EA9.0000000000000054

**Published:** 2024-06-04

**Authors:** Xavier Onrubia, Estefanía Martínez, Pedro Charco, Joana Baldó, Laura Reviriego, Robert Greif

**Affiliations:** From the Hospital Universitari Doctor Peset, València, Spain (XO, EM JB), Hospital Universitari i Politècnic La Fe, València, Spain (PC), Hospital Clínico Universitario, València, Spain (LR), University of Bern, Switzerland (RG) and School of Medicine, Sigmund Freud University Vienna, Vienna, Austria (RG)

## Abstract

**INTRODUCTION:**

Game-based learning, also called edutainment, has been promoted as an alternative to the unidirectional, passive teaching of traditional medical education. Solving enigmas and problems through creativity and critical thinking, which is encapsulated in ‘escape rooms’, has been adapted to teach medicine as a way to enhance the mental models of proceeding.

We considered an educational escape room as an activity to promote teaching and training in airway management, integrating knowledge, technical and nontechnical skills and collaborative teamwork during crisis situations.

**METHODS:**

No published experience on this topic was found. Therefore, we created an educational escape room focused on airway management.

We describe the steps undertaken from the design and development of the escape room process (as part of the curriculum of an international airway course) to the results of a survey completed by the participants at the end of the escape room process.

**RESULTS:**

Satisfaction with the experience was rated at least 8 points (0 to 10 numerical rating scale) by 80% of the 147 course participants. Two thirds also rated the experience as at least 8 points (0 to 10 Likert scale) as being helpful in improving behaviour in real cases, and enhance organisational teamwork skills.

**CONCLUSION:**

The airway escape room was feasible for training in airway management. Participants rated it as valuable to gain team competencies. The experience encourages further development and its possible use in other clinical settings.

**KEY POINTS**

- As part of game-based learning, educational escape rooms have become a growing alternative to traditional healthcare teaching.
- The time dedicated to the debriefing participants of an educational escape room experience must be a fundamental part to consolidate the objectives pursued with the activity.
- For an educational escape room to be useful, the target group and objectives must first be well defined, and from there, the activity is designed.
- The use of an airway escape room is an excellent opportunity to explore the development of both individual and team technical and nontechnical skills during airway management in different clinical scenarios, including stressful situations.

*“Tell me and I Forget,*

*Teach me and I Remember,*

*Involve me and I will Learn.”*

Benjamin Franklin, 1750

*“Having learned I start to change”*

Robert Greif, 2023

## Introduction

A recent innovation in medical education is experiential learning focused on reflection, critical analysis and synthesis of actions. Learners are actively engaged in investigation, questioning, experimenting, problem solving, taking on responsibility and constructing meaning out of the learning experience.^1^ One of these educational strategies is game-based learning, often also called ‘edutainment’, as an alternative to the unidirectional, passive teaching of traditional medical education.^2–4^

A current development in edutainment is the acquisition of new competencies based on the enhancement of mental models by solving enigmas and problems through creativity and critical thinking, which is encapsulated in ‘educational escape rooms’ adapted to teach medicine.^5^

In the early 2000s, escape rooms were created in Japan as entertainment, presenting series of puzzles that needed to be solved in a limited time with interconnected clues that finally allow the player or the team to leave the room.^6–9^ Players used their intellectual, creative and deductive reasoning skills to succeed. The activation of these cognitive mechanisms enhanced players’ abilities and team collaboration.

From an educational point of view, escape rooms bring together adult learning concepts like social constructivism, connectivism, emotional engagement and self-directed learning.^10^ By definition, an escape room is a participatory experience in which individuals are engaged in collaborative problem-solving, which can be used for educational purposes.^11^ Participants are inherently active learners often in an exciting, stimulating environment, aiming to enhance learning of different competencies and to foster their retention.^10,12,13^ Educational escape rooms are often designed for a specific target group with well defined learning goals,^9^ and have been developed in multiple healthcare disciplines (e.g. emergency medicine, pharmacy, nursing).^14^

This narrative report describes the development of an educational escape room in the field of airway management, aiming to combine technical and nontechnical skills, adherence to guidelines, and to enhance collaborative teamwork.

The delivery of the escape room experience was part of the interactive hands-on sessions during the 27th international airway management course from the International Training Group in Airway Teaching and Research (FIDIVA – Formación Internacional en Docencia e Investigación de Vía Aérea), in València, Spain in 2023. To our knowledge, this is the first description of an escape room in airway management. We also report results from the postcourse participants’ assessment of its utility and their learning satisfaction.

## Methods

### The challenge of designing an airway escape room

Initially, the possibility of developing an educational escape room focused on airway management as part of an international airway course (https://www.fidiva.com) was explored (EM, XO). After positive agreement experiences, airway educators (JB, PC, LR) joined the development of the airway escape room by connecting gaming ideas with learning goals, designing the tasks with a list of materials and possible problems to be solved. Finally, we created the organisational structure to link all the activities to guarantee a successful delivery.

### The planning phase: literature review, defining learning goals and devising a plot

A literature search in PubMed, Scopus and Google Scholar using the keywords, educational escape room, healthcare escape room and airway management did not bring up any article focused on airway management, only isolated reports in healthcare.^14^

**Figure 1:**
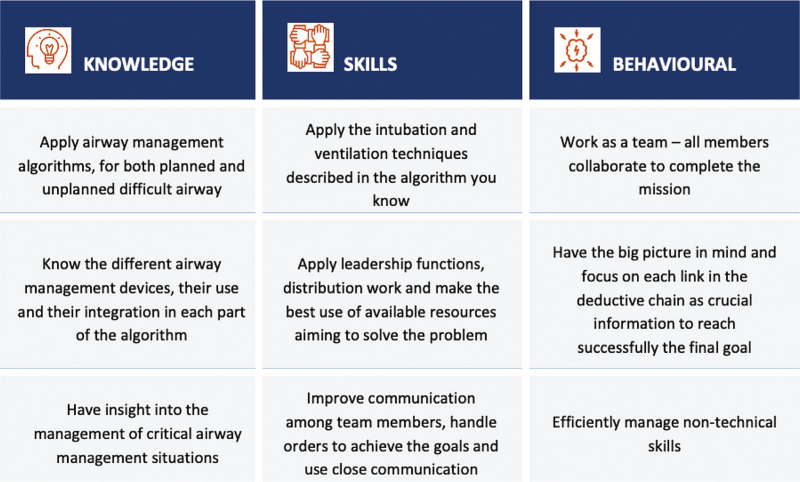
Programmed learning objectives of the airway escape room.

During an onsite brainstorming session and one online meeting, we shared and discussed ideas to achieve well defined learning objectives in terms of knowledge, technical and nontechnical skills and behaviour (Fig. 1). We explored the possibilities of transforming these objectives into suitable activities for an escape room, and designed a plot with the creation of clinical cases with the following assumption: the resolution of the cases requires information from the clinical history that would trigger the urgency of the interventions and the use of the most appropriate airway intervention (to be performed on an airway manikin). That should encourage discussion and teamwork between participants, who need to form, by themselves, teams solving each case.

We defined as objectives that participants of the escape room should adhere to current airway management algorithms, choose appropriate devices, and assign skilled persons to handle these, establish team competencies such as communication, collaboration and leadership, and finally apply contextualised information deductively to solve the given airway problem.

To trigger engagement in the escape room, the case vignette asked for the management of a critical airway problem in a virtual patient and the participants needed to ask for help via a phone call, but we planned to store all the participants’ mobile phones in a padlocked box. To retrieve the phones, a series of clinical cases and riddles providing clues to unlock the box had to be solved. Failure to decipher the padlock code meant that the group was not able to solve the critical airway problem, thus endangering the patient's life.

For organisational reasons of the course, in order to rotate everyone through the different workshop stations of the course, including the airway escape room, the total number of participants was randomly divided into 5 groups with an equal number of people in each group. However, this random distribution may have led to a lack of symmetry between the groups in terms of degree of experience (resident or not) and workplace (anaesthesiology, intensive care, emergency department).

### The preparation phase: from the plot to the staging and the design of the activity with the procurement of materials

Six airway management scenarios were designed. Each scenario consisted of a manikin, and to ensure that clinical decisions had to be made to solve the problem appropriately, several airway management devices were provided. Participants have to find the clues in the form of encrypted messages to solve the puzzles. The clues (e.g. letters, numbers, cards, tokens, QR codes) had to be correctly ordered to decode the locks to retrieve the participants’ mobile phones.

Our escape room was constructed with a hybrid structure,^7,9^ which combined an open structure (several puzzles can be solved at the same time) with a sequential structure (one after the other), and we used cognitive puzzles (requiring thinking and logic), as well as physical puzzles (requiring manipulation of devices; Fig. 2 ).^9^

**Figure 2:**
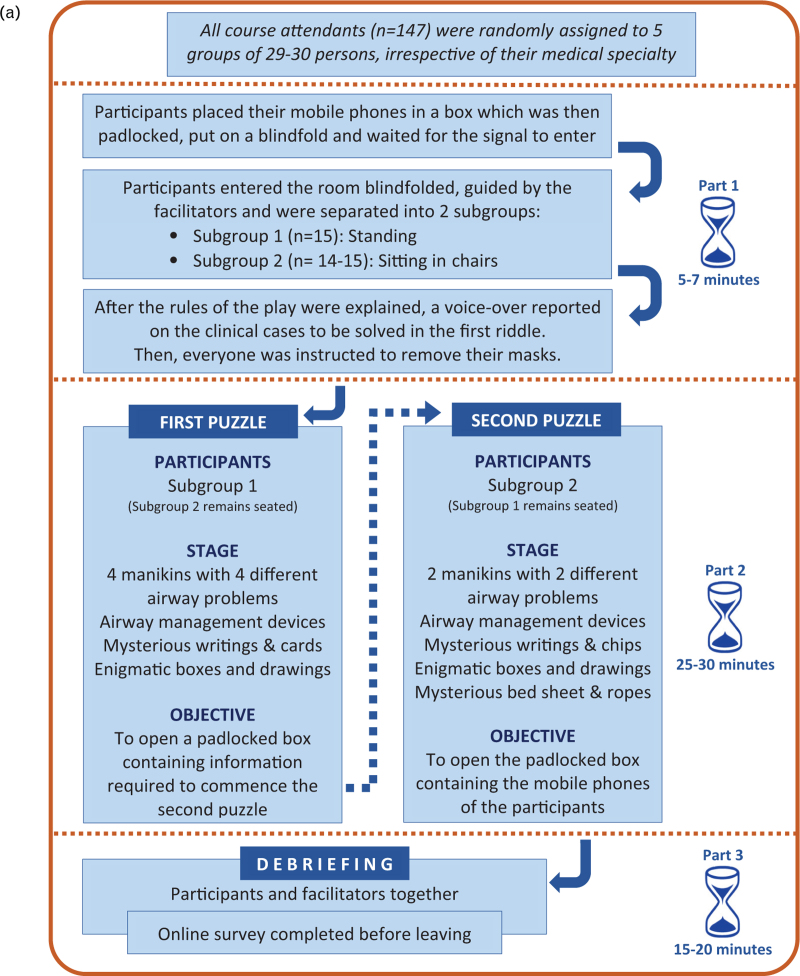
Development of the airway escape room (a). Blueprint of space and activities distribution (b).

**Figure 2 (Continued):**
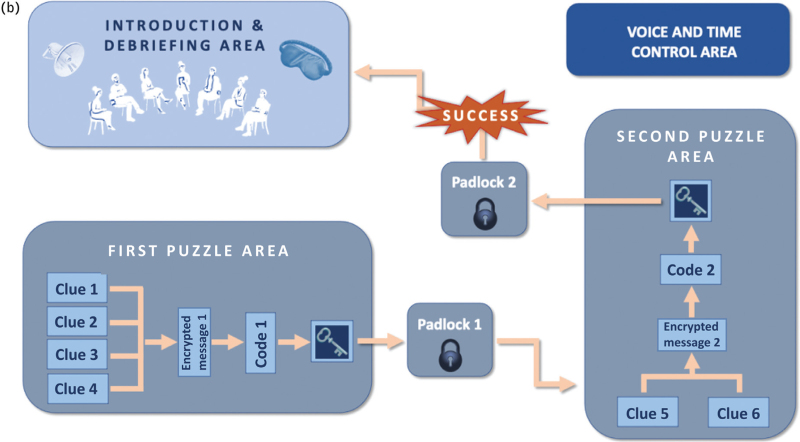
Development of the airway escape room (a). Blueprint of space and activities distribution (b).

We assumed that only if collaborative teamwork was established among the participants, would they be able to solve the puzzle on time. A voice-over of simulated progressive desaturation and bradycardia was created and programmed to conclude after 35 min (the maximum allotted time to solve the puzzles) to announce the beginning of the debriefing part. How the group handled the human factor side of airway management was planned to be the main focus, and this was to be emphasised in the debriefing, in line with Veldkamp conclusions.^9^

To fit the escape room with 30 participants into the 60 min time slots of the airway course, 55 min were set for each airway escape room session, with 5 min after the debriefing to check on the manikins and equipment and to tidy up and replenish used materials ready for the next session. To test the feasibility of the complex games, we assessed whether the activity could be carried out in a given time as the complexity of the game should allow success and not be too difficult for the participants acting as a team to complete.^10^ Test runs were recorded to extract improvements and to ensure an efficient and flowing process.

We considered three facilitators were needed to run an airway escape room. They were all familiar with the elements of each puzzle, which is important for assisting participants without compromising the challenge.^15^ All facilitators were disguised uniformly and wore Guy–Fawkes facemasks. To encourage the participants to buy-into the game, the walls of a standard lecture room were decorated with posters and materials related to airway management. Two external assistants were present to welcome participants, and to facilitate the organisational issues before the escape room experience started.

A list of required materials to undertake the activity was made, and three different sources of materials were considered:

(1) Those offered by the venue itself: a suitable room – about 70 m^2^ for the scenarios and a debriefing area (Fig. 2 ) – with tables, chairs, loudspeaker, computer, Wi-Fi and sufficient electrical connections.
(2) Those provided (without costs) by industry or the hospital: intubation heads, crico-trainers and sets, tracheal tubes, supraglottic devices, (video)-laryngoscopes, flexible bronchoscopes, ventilation bags, face masks and so forth.
(3) Those purchased and prepared by the escape room organisers: wooden boxes with a padlock, various cryptic writings, posters, labels, playing cards and tokens exclusively designed by the organisers and some QR codes (finding the mobile phone to decipher these codes was part of the game).

Each escape room session consisted of three parts (Fig. 2 ).

#### Part 1: introduction of participants to the airway escape room

Outside the room, all participants place their mobile phones in a box (which was then closed and padlocked) and then they put on randomly assigned blindfolds, some of which were marked with information useful for the game. The use of blindfolds had not been communicated to the participants. Then, participants were guided into the escape room and separated into two subgroups according to their type of blindfold (marked/unmarked). One subgroup stood and the other sat in chairs (Fig. 2 ). A voice-over introduced the theme and purpose of the airway escape room, and one facilitator explained the general rules of the game and how to ask for help. (Supplementary file ‘Appendix 1’). The entire group was invited to remove their masks and standing subgroup was invited to start.

#### Part 2: solving the puzzles, the performance phase

Four clinical airway cases were presented, and participants had to find the clues that provided information to handle the case with the most appropriate airway technique. Solving all four cases provided the combination to open the padlock of a box containing material to help solve the second puzzle of the escape room. Once this box was unlocked, the first subgroup was invited to sit whereas the second subgroup started with next part: two airway emergency cases with hidden clues from the medical history handled collaboratively as a group. Different materials found in the previous box were used to decipher the riddle, which enabled the last box containing participants’ mobile phones to be opened.

Participants needed to organise themselves into teams to solve the puzzles and clinical cases in each of the two parts. While one subgroup was performing, the other subgroup was observing the ongoing activity and interactions with the aim of collecting thoughts for the debriefing.

#### Part 3: debriefing

Strict time control for the two parts was essential to ensure enough time for debriefing, as this reflection phase enables deeper learning based on the planned learning objectives. Facilitators started the debriefing discussion with the obstacles encountered and how these were resolved by focusing on human factors like team collaboration, leadership, and communication under pressure and stress, and how that can be related to their clinical environment during airway management.

After the debriefing, participants were encouraged to fill in a survey (Supplementary file ‘Appendix 2’) via a mobile application (Google Forms) evaluating their experience with the airway escape room using a 0 to 10 numerical rating scale (0 = nil to 10 = excellent).

### The role of facilitators

Facilitators play an important role, which is challenging as students’ immersion and feelings of autonomy can be disrupted if too much intrusion happens.^9^ During the introduction part, the facilitators explained the game rules and learning goals. Throughout the problem-solving phase, participants’ autonomy in finding the solution was encouraged by guiding participants to follow known guidelines and to use the materials correctly. However, facilitators silently discouraged manoeuvres compromising the integrity of the set-up with gestures.^16,17^ To avoid participants’ frustration, a balance between helping and not helping them needed to be found, as providing hints to solutions too early hinders deductive self-learning,^18,19^ and not providing information when needed does not enable problem solving.^20^ Indirect clues were given by the facilitators if participants were far from achieving their goal and time was running out. Specific rules to ask for help were announced during the orientation at the introduction phase (Supplementary file ‘Appendix 1’).^19,21^

## Results

### First results from an escape room on airway management

During the FIDIVA airway course in Valencia, Spain in February 2023 (https://www.fidiva.com), all 147 registered participants were randomly assigned to five groups and each group participated in a different session of the airway escape room activity. Each group consisted of two subgroups of about 15 participants. One subgroup was engaged in the first task - solving four airway problems – to pass the clues to subgroup 2, which had to solve the remaining two emergency cases.

All groups participating in the airway escape room finished on time. In all of the five escape room sessions, facilitators were consulted for help to solve the puzzle. We observed that although everyone performed some tasks during the puzzle solving, some participants were not fully integrated or immersed into the game. That might be because of the relatively high number of participants limiting more active participation, or lack of competence in solving the airway case and allowing other participants to take the lead. The main complaint was the large group size, limiting face-to-face communication not easing team participation (30% of 46 comments on the escape room).

The survey was completed by 100% of the participants (Table 1). Overall participants expressed high satisfaction with the escape room and subjectively gained competencies, with high ratings especially for nontechnical skills (Table 2). Smaller groups and more upfront information about the escape room was suggested.

**Table 1:** Characteristics of the participants

	Specialist	Trainee	Total
Previous ER experience			57%
Sex (female/male)			147 (92/55)
Anaesthesiologist (female/male)	17/9	46/24	96 (63/33)
Intensive care (female/male)	6/6	19/10	41 (25/16)
Emergency (female/male)	4/6	–	10 (4/6)

Characteristics of the 147 participants. ER, escape room.

**Table 2:** Survey results

		0 to 4 rated	5 to 7 rated	8 to 10 rated
	Degree of satisfaction	7 (4.7)	22 (14.9)	118 (80.2)
The experience helped me to...	... improve behaviour in a real case	9 (6.1)	41 (27.8)	97 (65.9)
	… gain knowledge, skills or insight	10 (6.8)	40 (27.2)	97 (65.9)
	… improve organisational teamwork skills	9 (6.1)	40 (27.2)	98 (66.6)
Worked aspects	Teamwork	3 (2)	26 (17.6)	118 (80.2)
	Communication	6 (4)	28 (19)	113 (76.8)
	Adherence to algorithms	13 (8.8)	60 (40.8)	74 (50.3)
	Technical skills	27 (18.3)	65 (44.2)	55 (37.4)

Number of participants (%). Total 147 participants. Rating: 0 to 10 scale where 0 = none and 10 = very much.

## Discussion

We developed an airway escape room and implemented it during an international airway course (https://www.fidiva.com). Participants were satisfied and highly enjoyed the experience which encouraged collaborative teamwork and communication by following airway management algorithms. These highly rated results are in the frame of earlier reports of healthcare escape rooms.^22^

Our educational approach integrated role-playing simulation puzzles for small groups with technical and nontechnical airway skill challenges. The escape room format was designed in such a way that communication and teamwork were an essential part of solving the puzzles. Within the two main groups, the small groups of participants who had solved their cases offered help to other groups in solving their cases to achieve the common goal faster for the entire group of participants – getting their mobile phones back.

Each rotation group of 29 to 30 participants was randomly established, without following a pattern of distribution by speciality (workplace), by degree of experience (senior or residents) or by previous escape room experience. In this way, initiative was encouraged to enable the creation of small multidisciplinary groups to solve each clinical case. This model is used by Guckian to develop teamwork and cohesiveness^5^ and is considered to enable incorporation of different perspectives and allows division of tasks.^15^ In fact, some of these small groups, upon resolving their case, were willing to help those who had not yet resolved their assigned case.

Although previous escape room experience could have had an influence in solving the puzzles, we could not stratify the groups for that experience as we only obtained this information when participants completed the survey at the end of the escape room experience. As the groups were randomly created, having this information beforehand would not have changed the distribution of participants with this experience. In line with what we have mentioned before, one of the objectives of the activity was to promote collaborative teamwork and this includes a sum of individual behaviours, with technical and nontechnical skills, knowledge of and adherence to protocols and the experience of having previously participated in an escape room.

The differences between recreational and educational escape rooms are related to the setting where each takes place, time constraints of the game and the educational objectives, especially if life-saving skills are included (Supplementary file ‘Appendix 3’).^9^ However, for our educational purposes during a large airway event, adaptation was needed as described by others.^17,23^ Guigon *et al.*,^24^ for example, had two teams playing independently in the same escape room. Recreational escape rooms limit their participant numbers from three to six.^7^ As we had to deal with a large number of course participants, we assigned activities in two stages with two subgroups of 15 participants each and who acted sequentially in their teams to solve their task, aiming collaboratively to solve the overarching riddle similar as described earlier.^25,26^ To initiate leadership and teamwork, the fostering of collaborative participation, and immersion in the game, we opted for self-organising task-oriented small groups of four to six participants to solve the airway cases: such small group sizes strengthen cooperation,^27^ with better communication and organisation resulting in a shorter playing time.^9,16^ In addition, group sizes of up to six participants facilitate easier oversight by the instructors.^28^

Another substantial difference from a recreational escape room experience is the debriefing after the ‘excitement’, as reflection on the action is a major learning opportunity for the participants. After solving the riddles with all its excitement, a ‘cooling down’ time is needed from the intense interaction with each other while handling the cases.^7^ Sufficient time must be allowed to debrief the gameplay, to allow activation and decontextualization of knowledge, and finally to encourage personal reflection, and to provide guidance to the participants on how to translate what they have experienced and learned in the escape room to future clinical situations.^11^ Most students experienced that the debriefing improved clinical skills and facilitated learning.^29^ Due to our large group, we allocated about 30% of the escape room time for debriefing.^9^

One could think that the passive observational presence of the second subgroup during the first part of the game would help that subgroup to solve their part more easily and faster when it came to it. However, the first and second activities had completely different searches for information and resolution, so observation of one part did not offer any help to solve the other part.

The observations of both subgroups added important contributions to the debriefing discussions as an ‘outside’ view similar to a simulation session debriefing.^5^ In fact, during the debriefing, the conduct of both subgroups were explored and a process of reflection was initiated to learn which behaviours enabled success in solving the cases and puzzles. This is important to interrelate the main concepts and to take away doubts and incorrect ideas.^12^

The limitations of our escape room experience are a result of the large number of participants in each escape room session, the heterogeneity of medical specialties, language problems of an international event and the room we adapted for use was not specifically made for an escape room experience. We could only assess learning outcome on subjective impressions of the learners (Kirkpatrick's level 1). Whether participants of an escape room activity are able to transfer their experience and learning into their clinical practice is unknown, and this should trigger future research to ensure that the results justify the resources and time spent on an escape room educational format.

The survey was conducted anonymously, and the format was integrated into a mobile phone application (Google Forms) to collect the responses. Due to the design of the survey, it was not possible to analyse whether a respondent was a resident or a specialist, nor their workplace. Nor was it possible to analyse the responses by groups. Although such detailed analysis of this data was not intended for the current study, we are considering modifications of the form that would allow these data to be recorded and analysed.

## Conclusion

We describe the development and delivery of an airway management escape room as an educational activity at an airway management course. The extensive planning and preparation enabled us to run the escape room experience with five sequential groups, albeit each with a large number of participants. Ensuring that sufficient time is allowed for debriefing the participants after their escape room experience is important for consolidating learning. The postcourse questionnaire shows a high level of participants’ satisfaction, and the escape room experience was rated as very useful. These ratings encourage further development of escape room edutainment as an additional teaching tool in medical education for other specific interventions. Nevertheless, to justify the required resources and time, a thorough investigation is required to ascertain if the escape room experience translates into gained competencies in clinical practice.

## Supplementary Material

Supplemental Digital Content

## Supplementary Material

Supplemental Digital Content

## Supplementary Material

Supplemental Digital Content
