# Supplementary material for: New tools for learning airway management: A report on the creation of an escape room and its development during an international airway course
Source: Eur J Anaesthesiol Intensive Care. 2024 Jun 4;3(4):e0054. doi: 10.1097/EA9.0000000000000054 (PMC11798401; doi:10.1097/EA9.0000000000000054)
Supplement: Supplemental Digital Content [file ejaic-3-e0054-s001.docx]

**APPENDIX 1**

**Appendix 1. Rules for participants**

- Facilitators will not answer direct questions such as: W*hat should I look for*? or W*hat should I do*?

They will give only clues and indications they consider sufficient to facilitate the evolution of the process and the resolution of the enigmas.

- If at any time, the group is lost, not knowing how to proceed, an agreed sentence asking for help should be spoken jointly by all members of the group.
- When an action is totally nonsensical or may damage the equipment, the facilitator will prevent it without any explanation.
- The maximum time given to solve the puzzles and reach the final end point is 35 minutes. This time will be marked by an audible signal.
