# Supplementary material for: New tools for learning airway management: A report on the creation of an escape room and its development during an international airway course
Source: Eur J Anaesthesiol Intensive Care. 2024 Jun 4;3(4):e0054. doi: 10.1097/EA9.0000000000000054 (PMC11798401; doi:10.1097/EA9.0000000000000054)
Supplement: Supplemental Digital Content [file ejaic-3-e0054-s003.docx]

**APPENDIX 3**

**Appendix 3. Differences between recreational and educational escape rooms.**

| **Issue/Topic** | | **Recreational escape room** | **(Health care focused) educational escape room** |
| --- | --- | --- | --- |
| Audience | | Broad non-specific audience | Specific target group: health care professionals |
| Number of attendants | | Ideally up to 6 as a team | Large group (>10), several teams possible |
| Time | | No limit | Limited (course timetable) |
|  |  | Time constraint used to differentiate success or not | Time constraint used as part of goals in emergency scenarios |
| Setting | | One or more connected rooms | Usually a classroom or lecture hall |
|  |  | Permanent scenario | Temporarily customised for that purpose |
| Puzzles | Concept | Free and independent | Aligned with learning goals |
|  | Design | Any matter | Specific (focused on the specialty) |
| High Success rate | | Independent | Highly recommended |
| Debriefing | | Not necessary. | Essential: structured and goal directed |
